# Health Tract, No. 161

**Published:** 1865

**Authors:** 


### VICES OF GENIUS.

COLERIDGE was such a slave to liquor, that he had to be kept an unwitting prisoner by Christopher North on an occasion when some literary performance had to be completed by a certain time; and on that very day, without even taking leave of any member of the family, "he ran off at full speed down the avenue at Elleray, and was soon hidden, not in the groves of the valley, but in some obscene den, where, drinking among low companions, his magnificent mind was soon brought to a level with the vilest of the vile." When his spree was over, he would return to the society of decent men.

De Quincey was such a slave to the use of opium, that his daily allowance was of more importance than eating. "An ounce of laudanum a day prostrated animal life during the forenoon. It was no unfrequent sight to find him asleep on the rug before the fire in his own room, his head on a book, his arms crossed on his breast. When this torpor from the opium had passed away, he was ready for company about daylight. In order to show him off, his friends had to arrange their supper-parties so that, sitting until three or four in the morning, he might be brought to that point at which, in charm and power of conversation, he was so truly wonderful."

Burns was not less a drunkard than Coleridge. It was the weakness of Charles Lamb. And who can remember the last day of Poe without an irrepressible regret? He was on his way to marry a confiding woman, stopped in Baltimore, and was found, by a gentleman who knew him, in a state of beastly intoxication, unconscious as a log, and died that night in the ravings of *delirium tremens.*

Douglas Jerrold was a devotee of gin. Byron was a tippler, and his vile *Don Juan* was the inspiration of rum, as might well be supposed, for its indecencies make it unfit for any woman to read. Steele, "the brilliant author of the *Christian Hero*," was a beastly drunkard. Men wrote of him that "he would dress himself, kiss his wife and children, tell them a lie about his pressing engagements, heel it over to a groggery called 'The Store,' and have a revel with his bottle companions." Rollin says of Alexander the Great, that the true poison which brought him to his end was wine. The Empress Elizabeth of Russia was completely brutified by strong liquors. She was often in such a state of bacchic ecstasy during the day, that she could not be dressed in the morning; and her attendants would loosely attach some robes, which a few clips of the scissors would disengage in the evening.

Let every man, especially those in public life, who desires to avoid a drunkard's death, remember that he is on the crumbling verge of such an infamy when he begins to feel that in order to prepare himself, the doctor for a consultation, the lawyer for a cause, the clergyman for a sermon, the politician for a speech, he must take a pint of coffee, a cup of strong tea, a glass of brandy and water, or a plug of opium; and the self-same moment of that discovery let him put his foot down, raise his hand, and swear, that by the help of God he will never taste another grain or drop as long as life remains. This is the only safety.

